# Prognostic Value of Flow-Mediated Dilation and Reactive Hyperemia Index in Heart Failure: A Systematic Review and Meta-Analysis

**DOI:** 10.3390/jcm15010149

**Published:** 2025-12-24

**Authors:** Hanestya Oky Hermawan, Reny I’tishom, Meity Ardiana, Delvac Oceandy, Aida Fahira Rachma, Pratista Oktafia, Roy Bagus Kurniawan

**Affiliations:** 1 Doctoral Program of Medical Science, Faculty of Medicine, Universitas Airlangga, Surabaya 60132, Indonesia; hanestya.oky.hermawan.441193-2024@fk.unair.ac.id; 2 Department of Medical Biology, Faculty of Medicine, Universitas Airlangga, Surabaya 60132, Indonesia; ritishom@fk.unair.ac.id; 3 Department of Cardiology and Vascular Medicine, Dr. Soetomo General Hospital, Universitas Airlangga, Surabaya 60132, Indonesia; 4 Division of Cardiovascular Science, School of Medical Science, University of Manchester, Manchester M13 9PT, UK; delvac.oceandy@manchester.ac.uk; 5 Faculty of Medicine, Universitas Airlangga, Surabaya 60132, Indonesia; aidafahira.r@gmail.com (A.F.R.); pratistaoktafia31@gmail.com (P.O.); roykurniawan72@gmail.com (R.B.K.)

**Keywords:** flow-mediated dilation, reactive hyperemia index, endothelial function, heart failure

## Abstract

**Background/Objectives**: There is a growing acknowledgment of the role of endothelial dysfunction as an outcome predictor and therapeutic target in heart failure (HF). Flow-mediated dilation (FMD) and the reactive hyperemia index (RHI) are non-invasive diagnostic measures of endothelial dysfunction. In this meta-analysis, we aimed to highlight the importance of endothelial dysfunction, as measured by FMD and RHI, and its association with clinical outcomes, including mortality, hospitalization, and exercise capacity in patients with HF. **Methods**: We reviewed observational studies assessing clinical outcomes of HF patients with and without impaired FMD and/or RHI. Searches around electronic research databases were conducted using predetermined keywords. Meta-analysis was subsequently performed on selected studies that assessed adverse events in patients with HF. Our primary outcome was adverse events, which include mortality, disease progression, hospitalization, and complications (stroke, myocardial infarction, and heart attack). **Results**: This review included 16 studies, with a total of 1890 participants. Meta-analysis demonstrated that lower FMD in patients with HF had a significantly higher risk of adverse events (HR 1.44, 95% CI 1.15–1.82; *p* = 0.002; I^2^ = 80%, random-effects model). In contrast, lower RHI was not associated with an increased risk of adverse events (HR 1.32, 95% CI 0.96–1.82; *p* = 0.09; I^2^ = 87%, random-effects model). **Conclusions**: Endothelial dysfunction is associated with adverse endpoints in HF. FMD showed consistent prognostic values, while RHI’s prognostic significance is less clear and requires further investigation.

## 1. Introduction

There is a growing acknowledgment of the role of endothelial dysfunction in predicting cardiovascular events related to heart failure (HF). Endothelial dysfunction is a shift from a normal state to a pro-inflammatory, prothrombotic condition in the endothelial layer of arteries, veins, and capillaries. Endothelial dysfunction is closely linked to oxidative stress, chronic inflammation, and dysregulation of nitric oxide (NO), which further contribute to vascular stiffness and impaired perfusion [1]. A previous study reported that endothelial dysfunction is linked to disease progression in chronic heart failure (CHF), highlighting its impact on increased cardiac afterload due to systemic and pulmonary vasoconstriction, as well as alterations in vasomotor function [2]. Flow-mediated dilation (FMD) and the reactive hyperemia index (RHI) are non-invasive diagnostic measures of endothelial dysfunction [3]. While FMD has been extensively studied in the CHF population, evidence for the RHI remains limited [4].

FMD assesses endothelial function using ultrasound by measuring the percentage of change between brachial artery diameter during dilation (also termed reactive hyperemia) after 5 min of occlusion compared to baseline diameter. In contrast, the RHI reflects microvascular dilation, typically measured at the fingertip using reactive hyperemia–peripheral arterial tonometry (RH-PAT) before and after occlusion [3,5]. Impaired FMD has been observed in numerous conditions, including hypertension, diabetes mellitus, coronary artery disease, chronic kidney disease, systemic infection and sepsis, autoimmune diseases, and active cancer undergoing chemotherapy [6,7,8].

A previous study reported the role of FMD in predicting unfavorable outcomes in HF patients, such as heart transplantation, left ventricular assist device (LVAD) implantation, and cardiac death [9]. Impaired FMD is also predictive of the progression of HF [10]. Meanwhile, another study also found that low RHI is correlated with the incidences of mortality and hospitalization [4]. Therefore, we hypothesize that FMD and RHI may have prognostic value as risk stratification tools and may improve outcome prediction for HF patients when used alongside traditional risk factors.

In addition, although recent FMD studies use semi-automated software, the procedure remains operator-dependent and requires a skilled technician [5,11]. To minimize the variability of test results, expert consensus had been established for the FMD protocol [12,13]. Meanwhile, RHI is measured through RH-PAT, an emerging non-invasive method that, unlike FMD, does not require a skilled technician [1].

FMD and RHI assess endothelial function through different vascular territories and mechanisms; comparing them provides a more comprehensive understanding of endothelial dysfunction and its prognostic value in heart failure. To the best of our knowledge, there is no previous meta-analysis that assessed FMD and RHI as predictive tools for HF outcome. Therefore, in this review, we aim to highlight the importance of endothelial dysfunction, as measured by FMD and RHI, and its association with clinical outcomes in patients with HF (e.g., mortality, hospitalization, and exercise capacity).

## 2. Materials and Methods

This review followed the 2020 Preferred Reporting Items for Systematic Reviews and Meta-Analyses (PRISMA) guidelines [14]. Details of the PRISMA checklist and checklist for abstract are available in Supplementary Tables S1 and S2. The protocol for this review was registered in PROSPERO under the registry number CRD420251077312.

### 2.1. Eligibility Criteria

Eligible studies for this review must meet the following criteria: (i) observational studies on the human population with HF; (ii) assessment of FMD and/or RHI; (iii) observing the clinical outcomes, such as cardiovascular events, mortality, hospitalization, quality of life, and exercise tolerance; (iv) FMD measured by calculating the percentage change in arterial diameter using ultrasound; and (v) RHI measured using RH-PAT. Further inclusion criteria for this review required the study population to have stable HF, regardless of the type of ejection fraction (EF) and measurement of FMD using ultrasound, as well as RHI using RH-PAT. The exclusion criteria were (i) patients with acute HF or acute decompensated HF; (ii) individuals with comorbidities that could confound endothelial function (e.g., active cancer, autoimmune diseases, systemic infections); (iii) pregnant women; and (iv) additional interventions that may confound endothelial function.

### 2.2. Search Strategy

The study search was conducted on research databases such as Scopus, ScienceDirect, Proquest, EBSCO, IEEE, DOAJ, PubMed, Web of Science, and Cochrane. Keywords used for the search were predetermined as “flow-mediated dilation” OR “flow-mediated vasodilation” OR “endothelial function” AND “heart failure” OR “chronic heart failure” OR “HFpEF” OR “HFrEF” AND “hospitalization” OR “mortality” OR “quality of life” OR “outcomes” AND “patients” OR “subjects”. The detailed search strategy in each database is provided in Supplementary Table S3. Search results were extracted and uploaded to the Rayyan (https://rayyan.ai/reviews, accessed on 20 June 2025) database. Studies were screened independently by two reviewers, with any discrepancies resolved through consultation with a third reviewer. After the screening process, the full text of every article potentially eligible for inclusion was retrieved.

### 2.3. Data Extraction

General data from the included articles were extracted and appraised, including author(s), article title, study location (country), publication year, and study design. Further appraisal was conducted regarding the methodology of FMD and RHI assessment, the discontinuation of possible confounding drugs and other vasodilators, as well as exercise before the assessment, and additional intervention/exposure if available. Lastly, the outcomes of each article were appraised qualitatively and quantitatively.

### 2.4. Risk of Bias Assessment

The included study was assessed for the risk of methodological bias using the Newcastle–Ottawa Scale (NOS) for cohort and case–control studies, as well as the modified NOS adapted to assess cross-sectional studies [15,16]. This process was carried out independently by two reviewers and discrepancies were resolved through further discussion.

### 2.5. Statistical Analysis

Effect measures for each study outcome were extracted and grouped accordingly. The primary effect measure was the hazard ratio (HR). When HRs and their corresponding confidence intervals were directly reported in the included studies, these values were extracted. For studies that did not provide HRs explicitly, indirect HRs were calculated using the method previously described by Hebert et al. (2022) [17]. A meta-analysis was performed using the Review Manager (RevMan, version 5.4., Cochrane Collaboration, London, UK) software to generate pooled effect estimates. Pooled HRs with 95% confidence intervals (CIs) were calculated using the inverse variance method. Statistical heterogeneity among studies was assessed using Cochran’s Q test (chi-square test) and quantified using the Higgins I^2^ statistic, which describes the percentage of total variation across studies due to heterogeneity rather than chance. An I^2^ value of 25%, 50%, and 75% was considered low, moderate, and high heterogeneity, respectively.

In the presence of low heterogeneity (I^2^ < 50%), a fixed-effect model was applied; otherwise, a random-effects model (DerSimonian–Laird method) was used to account for between-study variability. Sensitivity analyses were planned by sequentially excluding individual studies to evaluate the robustness of the pooled results. Publication bias was explored visually through funnel plots if ≥10 studies were available for one of each outcome.

## 3. Results

### 3.1. Study Selection Process

**Figure 1:**
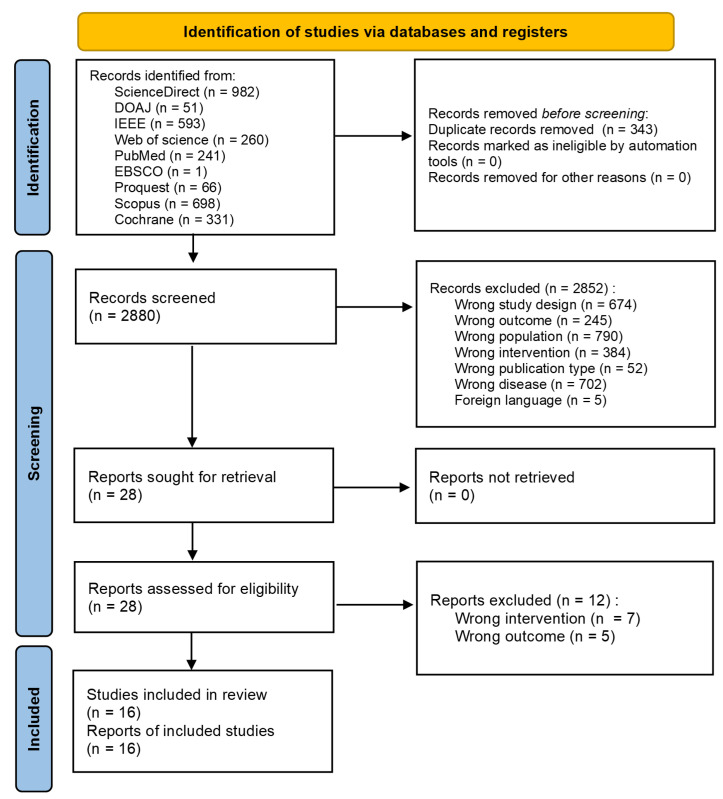
PRISMA flow diagram.

We retrieved a total of 3223 studies across databases. Abstract screening was performed to exclude studies according to the predetermined criteria. The detailed process is described in the PRISMA flow diagram (Figure 1).

### 3.2. Characteristics of Included Studies

This review included 16 studies enrolling a total of 1890 participants. Each study characteristic was presented in Table 1. All studies underwent risk of bias assessment: 14 cohort studies were classified as low risk, one cross-sectional study was rated as low risk of bias, and another cross-sectional study was assessed as moderate risk of bias (Tables S4 and S5). Table 2 summarizes population characteristics and effect estimates for studies included in the meta-analysis.

### 3.3. Adverse Events

Adverse events including mortality, disease progression, hospitalization, and complications (stroke, myocardial infarction, and heart attack) were the primary outcome assessed in this review. Meta-analysis was performed, including only studies assessing FMD in the HF population with ejection fraction (EF) < 50%. Due to the limitation of the number of eligible studies, we did not perform meta-analysis for the population of HF with preserved ejection fraction (HFpEF). Indirect HRs for studies by Meyer et al. (2005), and Shechter et al. (2009) [20,25] were calculated using the method previously described by Hebert et al. (2022) [17]. The calculation can be found in Appendix A (Table A1).

**Figure 2:**
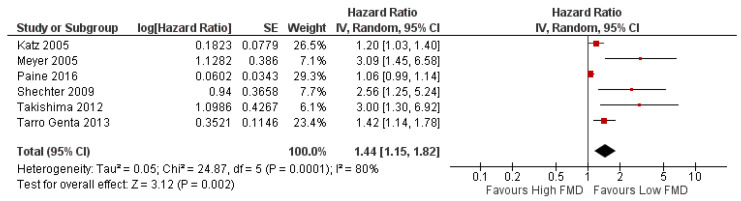
Forest plot of prognostic value of FMD to adverse events in HF patients with EF < 50% [4,9,10,20,24,25]. In the forest plot, the red box represents the hazard ratio for each study, the horizontal line shows the confidence interval, and the black diamond represents the pooled hazard ratio from all studies.

The heterogeneity test result of the studies included in the meta-analysis revealed a significant difference; therefore, a random effect analysis was performed. The test for overall effect summarized that a significant risk of adverse events was observed in HF patients with EF < 50% presenting with lower FMD (HR 1.44 [95% CI 1.15–1.82], *p* = 0.002, I^2^ = 80%, random effect) (Figure 2). A leave-one-out sensitivity analysis was performed (Figures S1–S6), and no significant change in heterogeneity was observed. Because only six eligible studies were available for this outcome (fewer than the recommended minimum of ten), a funnel plot was not generated.

**Figure 3:**
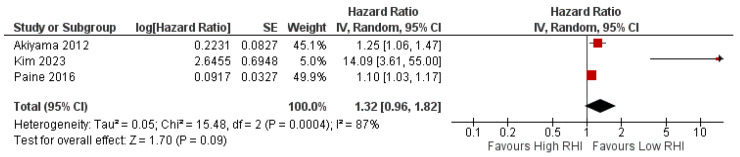
Forest plot of prognostic value of RHI to adverse events in HF patients [4,26,28]. In the forest plot, the red box represents the hazard ratio for each study, the horizontal line shows the confidence interval, and the black diamond represents the pooled hazard ratio from all studies.

Analysis was also conducted for studies examining the prognostic value of RHI in relation to adverse events in HF patients. However, we included all studies assessing the general HF population without differentiating the ejection fraction classifications, due to the limited number of studies. The result for the heterogeneity test was also significant—therefore, a random-effect model was applied. The overall test result showed a non-significant risk of adverse events in HF patients presenting with lower RHI (HR 1.32 [95% CI 0.96–1.82], *p* = 0.03, I^2^ = 87%, random effect) (Figure 3). A leave-one-out sensitivity analysis was also performed (Figures S7–S9). There was a significant heterogeneity change after leaving out the study by Kim et al. (2023) [28]. A funnel plot was not generated as the number of studies did not meet the minimum criteria.

### 3.4. Mortality and Hospitalization

Numerous studies have examined the predictive significance of endothelial function in individuals with HF. Kim et al. (2023) [28] found that in a group of 90 patients who had been hospitalized for HF before, a low reactive hyperemia index (RHI < 1.48) was strongly linked to a higher risk of bad clinical events, such as death and repeated hospitalization. Patients in the low RHI group had a risk that was up to 14 times higher [28]. Yufu et al. (2015) [30] showed that in 34 patients with advanced HF who were receiving cardiac resynchronization therapy (CRT), a lower RHI (≤1.5) was an independent predictor of rehospitalization due to HF progression. All the deaths observed occurred within this group [30]. In addition to these results, Shechter et al. (2009) conducted a prospective study on 114 patients with chronic HF and reduced LVEF (<35%), demonstrating that poor endothelial function, as measured by FMD (<7.1%), independently predicted both rehospitalization and death [25].

### 3.5. Exercise Capacity

FMD was observed as the predictor of exercise capacity in a few studies. In a cross-sectional study by Vittorio et al. (2010) [18], patients aged 25–71 years with HFrEF underwent cardiopulmonary exercise testing (CPET). There was a moderate relationship between FMD and % maximum predicted heart rate (MPHR) and a low-strength relationship between FMD and peak oxygen consumption (peak VO_2_) [18]. Meanwhile, a cross-sectional by Haykowsky et al. (2012) [22], which assessed elderly HFpEF patients, reported a modest relationship between FMD and peak VO_2_. However, after adjustment for other variables including age, the study reported no significant relationship [22]. Two cohorts also investigated the relationship between FMD and 6 min walking test (6MWT) results. In adults with HF receiving CRT, FMD improvement 3 months post-implantation was found to be significantly correlated with 6MWT improvement [19]. In comparison, individuals with HFrEF (EF ≤ 35%) who underwent 3 months of optimal medical therapy demonstrated significant improvements in FMD, which were strongly correlated with enhancements in the 6MWT [23].

## 4. Discussion

Several established prognostic factors for HF outcomes have been widely reported, such as older age, ejection fraction, NYHA class, BNP, NT-pro-BNP, and creatinine levels [31,32]. In addition, regardless of subtypes and the presence of atrial fibrillation, the CHA2DS2-VASc score had also been predictive of HF outcome [33,34]. FMD, a non-invasive measure of endothelial function, has been investigated for its prognostic value in predicting cardiovascular events. Endothelial dysfunction, as reflected by impaired FMD and/or RHI, is linked to the progression of cardiovascular disease and mortality [35]. Therefore, its prognostic value may extend to special populations, such as HF patients. There is emerging evidence supporting the use of FMD and RHI as a prognostic tool in the HF population [4,9,10,20,24,25,27,29].

Although the two measures appear similar, it is important to note that the Framingham Heart Study reported that FMD and RHI assess distinct values. The study suggested that the RHI is more affected by metabolic factors, whereas FMD is highly affected by age [36]. The difference in FMD and RHI may be due to several reasons: (1) FMD is dependent on NO, meanwhile RH-PAT only partly relies on it—further explaining why FMD is more strongly associated with age [37]; (2) RH-PAT, which measures finger microvasculature, may exhibit different physiological responses to ischemia due to the dual circulation consisting both capillaries and arteriovenous anastomoses [36,38]; and (3) it is also hypothesized that physiological processes may preserve distal vessel hyperemic responses [36,38].

Our main findings show that FMD is associated with adverse outcomes in HF patients with EF < 50%, whereas RHI is not significantly linked to adverse outcomes in the general HF population (Figure 2 and Figure 3). The FMD meta-analysis included populations with an ejection fraction <50%, without differentiating between HFrEF and HF with mildly reduced EF (HFmrEF). This decision was made because HFmrEF was introduced in 2016 [39]; meanwhile, the majority of the included studies predated this classification. The meta-analysis on FMD and RHI prognostic value showed a significant heterogeneity. There are several explanations for this: (1) different methods were used for HR estimation—while a few studies used dichotomized HR, the remaining studies used continuous HR; (2) there were differences in adjustment for confounders across studies; and (3) there were differences in FMD threshold or cut-off (Table 2). Another possible explanation is the difference in the FMD measurement protocol. There is a noticeable difference in the measurement of brachial artery diameter post-cuff deflation, with several studies using maximum dilation measured between 30 s before and 120 s after deflation, while other studies measure dilation at 60 s after deflation. Both of these methods were previously described in the literature [12,13]. However, recent expert consensus has argued that this timing may underestimate true peak brachial artery diameter, and thus recommends measurement up to 180 s post cuff deflation [13].

Our study also highlighted that lower FMD and RHI are prognostic for the incidence of hospitalization and mortality in HF patients [25,28,30]. These studies demonstrate that endothelial dysfunction, indicated by diminished RHI or FMD, is consistently linked to elevated risks of rehospitalization and mortality. This endorses their potential utility as non-invasive biomarkers for risk stratification in HF. In addition, FMD is also found to correlate with exercise capacity, indexed by peak VO_2_ and MPHR. Improvements in FMD are likewise associated with improvements in the 6MWT [18,19,22,23].

Endothelial dysfunction, indicated by reduced FMD, plays a key role in the development and progression of cardiovascular diseases, including HF. In patients with HFrEF, this dysfunction is driven by neurohormonal activation, altered shear stress, and NO dysregulation [40]. This dysregulation subsequently decreases endothelium-dependent vasodilation and coronary blood flow, causing reduced myocardial perfusion, which worsens ventricular function [40,41]. NO imbalances also affect matrix metalloproteinases, which further cause cell migration, cardiac hypertrophy, and atherosclerotic plaque stability [41].

Since FMD is a more well-established tool for assessing endothelial function, many studies have used it to validate RH-PAT. However, differing results have been reported between studies [36,42]. Despite the differences between FMD and RHI, previous research has reported that RHI remains a reliable method for assessing endothelial dysfunction in adults with HF [43]. Another relevant study worth mentioning is a cohort by Matsue et al. (2013) [44], which showed that log-transformed RHI has a prognostic value on HFpEF. Endothelial dysfunction played a role in the development of HFpEF, although it is not the only pathomechanism contributing to the decline in cardiovascular function [44,45].

Many studies have repeatedly shown that endothelial dysfunction is a key factor in the pathogenesis of HFpEF, with reduced FMD being one of its main signs. Lee et al. (2016) [46] demonstrated that individuals with HFpEF exhibited a markedly diminished brachial artery FMD. However, a portion of the impairment might be accounted for by reduced shear stress, which shows that microvascular dysfunction is more important than conduit artery failure [46]. Kishimoto et al. (2021) further substantiated the diminished FMD in HFpEF, indicating that the impairment is associated with increased arterial stiffness and systemic vascular remodeling [47]. In a larger cohort, Merechaux et al. (2016) and Ambrosino et al. (2021) showed that reduced FMD in HFpEF was significantly correlated with diminished exercise capacity and diastolic dysfunction [48,49]. A new analysis has confirmed that FMD independently forecasts negative outcomes in HF across ejection fraction subtypes and signifies a prospective therapeutic target [50].

These findings have driven the investigation into treatments for endothelial dysfunction in cardiovascular and metabolic diseases. Shigiyama et al. (2017) demonstrated that in a population with type 2 diabetes, the use of dapagliflozin significantly improved FMD compared to the control group [51]. Contrary to this study, Zainordin et al. (2016) found that dapagliflozin treatment in patients with type 2 diabetes and ischemic heart disease showed no significant difference in FMD between the placebo and treatment groups [52]. Since 2015, increasing attention has been given to the use of sodium-glucose transport 2 (SGLT-2) inhibitors in HF [53,54], particularly in HFpEF, since previous therapies had been less effective [55,56]. A recent trial had also discussed that SGLT-2 inhibitor, particularly empagliflozin, was associated with improvement in endothelial function measured with FMD in the population with CHF and type 2 diabetes [57]. However, additional studies involving larger populations are needed to establish stronger evidence.

### 4.1. Study Limitation

Certain limitations of the current research must be acknowledged. The number of available studies is limited, particularly those evaluating RHI, hence reducing the overall strength of the evidence. Numerous studies were conducted with relatively small sample sizes, indicating the possibility of publication bias, as favorable outcomes are published more frequently than adverse or neutral results. Moreover, the heterogeneity in methodology for assessing endothelial function, differences in the studied populations (e.g., HFrEF, HFpEF, or post-CRT cohorts), and inconsistencies in the definitions of clinical outcomes restrict the generalizability of these findings.

### 4.2. Future Research Recommendation

For future research, additional prospective studies are necessary to explicitly evaluate the prognostic importance of FMD and RHI across different subtypes of HF. Standardizing the methods used to measure endothelial function is important for ensuring that results from different studies can be compared and replicated. Subsequent research should explore the integration of endothelial biomarkers with established clinical characteristics and additional biomarkers, such as NT-proBNP or cardiac imaging, to enhance the accuracy of risk stratification. A comprehensive technique may ultimately solidify endothelial function testing as an essential component of prognostic assessment in HF.

## 5. Conclusions

Endothelial dysfunction is associated with adverse endpoints in HF, including rehospitalization, mortality, and impaired exercise capacity. FMD showed consistent prognostic value, particularly in HFrEF, and serves as a promising non-invasive biomarker for risk stratification and monitoring patient functional status. Meanwhile, the prognostic significance of RHI is less clear and requires further investigation. Integrating endothelial function evaluation into clinical practice could improve the identification of high-risk patients and guide optimal therapy, particularly as a non-invasive biomarker.

## Figures and Tables

**Table 1:** Characteristics of included studies.

No	Author (Year)	Country	Study Design	Sample Size	Population	Measurement	Key Findings	Quality Assessment
1	Vittorio et al. (2010) [18]	USA	cross-sectional	30	CHF patients on optimal beta-blocker and ACE inhibitor treatment	FMD	FMD and MPHR moderately correlated in HF patients receiving OMT.	Low Risk of Bias ^a^
2	Akar et al. (2008) [19]	USA	cohort prospective	33	HFpEF undergoing CRT implantation	FMD	Lower baseline FMD was correlated with the distance achieved on 6MWT prior to CRT implantation. Improvement in FMD correlated with improvement in 6MWT post CRT implantation.	Low risk of bias ^b^
3	Tarro Genta et al. (2013) [9]	Italy	cohort prospective	71	HF patients on stable OMT	FMD	FMD, PSR, and normalized FMD were predictors for heart transplantation, LVAD implantation, and cardiac death.	Low risk of bias ^b^
4	Meyer et al. (2005) [20]	Austria	cohort prospective	75	CHF patients UNOS status 2 despite OMT	FMD	FMD, log BNP, and mean blood pressure were independently related to the combined endpoints including conversion to UNOS status 1 and death.	Low risk of bias ^b^
5	Xie et al. (2016) [21]	China	cohort prospective	52	HF due to idiopathic dilated cardiomyopathy on beta-blocker therapy	FMD	Baseline FMD is a significant independent predictor of changes in LVEF and LVRR in IDC patients treated with beta blocker.	Low risk of bias ^b^
6	Takishima et al. (2012) [10]	Japan	cohort prospective	245	Stable ischemic CHF with impaired FMD < 5.5% at initial measurement	FMD	Persistent impairment of FMD was an independent predictor of cardiac death and decompensating of heart failure.	Low risk of bias ^b^
7	Paine et al. (2016) [4]	USA	cohort prospective	156	CHF	FMD, RHI	Reduced RHI was predictive of death or cardiac hospitalization, while FMD was not predictive.	Low risk of bias ^b^
8	Haykowsky et al. (2012) [22]	USA	cross-sectional	113	66 older HFpEF patients, 31 older healthy participants, 16 young healthy participants	FMD	There was no significant relationship between brachial artery FMD and peak VO_2_ in elderly HFpEF patients.	Moderate risk of bias ^a^
9	Poelzl et al. (2006) [23]	Ireland	cohort prospective	33	HFpEF patients on OMT consisting of beta blockers and ACE inhibitor	FMD	FMD were directly correlated with increases in 6MWT in the population studied.	Low risk of bias ^b^
10	Katz et al. (2005) [24]	USA	cohort prospective	149	CHF	FMD	Decreased FMD was significantly associated with increased mortality risk in ischemic and non-ischemic CHF patients.	Low risk of bias ^b^
11	Shechter et al. (2009) [25]	Israel	cohort prospective	82	CHF	FMD	Decreased FMD is significantly associated with increased mortality, hospitalization due to CHF exacerbation, and acute myocardial infarction.	Low risk of bias ^b^
12	Akiyama et al. (2012) [26]	Japan	cohort prospective	320	Stable HF	RHI	RHI is an independent predictor for future cardiovascular events in patients with HFpEF.	Low risk of bias ^b^
13	Fischer et al. (2004) [27]	Germany	cohort prospective	67	CHF	FMD	Radial FMD is independently associated with an increase in mortality due to cardiac causes, heart transplantation, and hospitalization.	Low risk of bias ^b^
14	Kim et al. (2023) [28]	Korea	cohort prospective	90	CHF with history of hospitalization	RHI	A low RHI value was associated with an increased risk of clinical events, including all-cause mortality and HF readmission.	Low risk of bias ^b^
15	Tsigkou et al. (2023) [29]	Greece	cohort prospective	340	CHF	FMD	Decreased FMD levels are associated with the incidence of MACEs during follow-up in patients with ischemic HF.	Low risk of bias ^b^
16	Yufu et al. (2015) [30]	Japan	cohort prospective	34	CHF with CRT	RHI	Baseline RHI value was independently associated with the incidence of new hospitalization due to HF progression.	Low risk of bias ^b^

Abbreviations: 6MWT = 6 min walking test; ACE = Angiotensin-converting enzyme; BNP = B-type natriuretic peptide; CHF = chronic heart failure; CRT = cardiac resynchronization therapy; EF = ejection fraction; FMD = flow-mediated dilation; HF = Heart failure; HFpEF = HF with preserved ejection fraction; HFrEF = HF with reduced ejection fraction; LV = left ventricle; LVAD = LV assist device; LVEF = LV ejection fraction; LVRR = LV reverse remodeling; MACEs = Major adverse cardiac events; MPHR = Maximal predicted heart rate; N/A = Not available; NYHA = New York Heart Association; OMT = optimal medical therapy; Peak VO_2_ = peak oxygen consumption; PSR = peak shear rate; RHI = reactive hyperemia index; UNOS = United network of organ sharing; USA = United States of America. ^a^ Risk of bias assessment using adaptation of the NOS for cross-sectional studies. ^b^ Risk of bias assessment using NOS for cohort studies.

**Table 2:** Population characteristics and effect estimates of studies included in the meta-analysis.

No	Author (Year)	Inclusion Criteria			Measurement	Primary Outcome	FMD/RHI Cut-Off	Effect Estimate	Adjusted Variables
		HF Etiology	EF	NYHA					
1	Katz et al. (2005) [24]	N/A	≤40%	II–III	FMD	Adverse events (death, urgent transplantation)	1.79%	HR estimate for a 1% decrease in FMD = 1.20, 95% CI 1.03–1.45, *p* = 0.027; Dichotomized HR = 3.45, 95% CI 1.10–11.1, *p* = 0.0033	Age, etiology of CHF, NYHA class, LVEF
2	Shechter et al. (2009) [25]	Ischemic CHF	≤30%	IV	FMD	Adverse events (mortality, non-fatal myocardial infarction, and hospitalization for CHF exacerbation)	4.6%	N/A	N/A
3	Tarro Genta et al. (2013) [9]	Ischemic or idiopathic dilated cardiomyopathy	<40%	≥II	FMD	Adverse events (mortality, LVAD implantation, heart transplant)	N/A	HR = 0.703, 95% CI: 0.547–0.904, *p* < 0.006	PSR
4	Meyer et al. (2005) [20]	N/A	≤30%	N/A	FMD	Adverse events (mortality, UNOS status 1 for heart transplant)	6.8%	N/A	N/A
5	Takishima et al. (2012) [10]	Ischemic	<50%	II or III	FMD	Adverse events (cardiac death or hospitalization due to decompensation of HF)	5.5%	Dichotomized HR = 3.0, 95% CI 1.3–6.9, *p* = 0.013	Age, hypertension, heart rate, NYHA III, hemoglobin, serum sodium, HbA1c, BNP, FMD, eGFR
6	Paine et al. (2016) [4]	N/A	≤40%	II or III	FMD	Adverse events (mortality or cardiac hospitalization)	N/A	HR = 1.062; 95% CI 0.993–1.135, *p* = 0.08	HF etiology, LVEF, NT-ProBNP, age, baseline heart rate, baseline arterial diameter, hyperemic flow, hypercholesterolemia
					RHI	Adverse events (mortality or cardiac hospitalization)	High > 0.975 L/min; Intermediate = 0.64–0.975 L/min; Low < 0.64 L/min	HR = 0.912 (0.854–0.973) *p* = 0.0055	
7	Kim et al. (2023) [28]	N/A	N/A	II–III	RHI	Mortality and HF readmission	1.48	HR = 14.09; 95% CI 3.61–54.99; *p* < 0.001	Age, sex, BMI, hypertension, diabetes mellitus, eGFR, LVEF, RAS blocker
8	Yufu et al. (2015) [30]	N/A	<35%	III–IV	RHI	Cardiovascular events	N/A	HR = 0.80; 95% CI 0.67–0.94, *p* = 0.007	Log BNP, baseline RHI

Abbreviations: BNP = B-type natriuretic peptide; BMI = body mass index; CHF = chronic heart failure; CI = confidence interval; CRT = cardiac resynchronization therapy; EF = ejection fraction; eGFR = estimated Glomerular Filtration Rate; FMD = flow-mediated dilation; HF = heart failure; HR = hazard ratio; LVAD = left ventricular assist device; LVEF = LV ejection fraction; N/A = Not available; NT-proBNP = N-terminal pro-brain natriuretic peptide; NYHA = New York Heart Association; PSR = peak shear rate; RAS = Renin–Angiotensin–Aldosterone; RHI = reactive hyperemia index; UNOS = United network of organ sharing.

## Data Availability

The original contributions presented in this study are included in the article/Supplementary Material. Further inquiries can be directed to the corresponding author.

## Supplementary Materials

The following supporting information can be downloaded at https://www.mdpi.com/article/10.3390/jcm15010149/s1, Table S1: PRISMA Checklist (2020); Table S2: PRISMA Checklist for Abstract (2020); Table S3: Detailed search strategy for each database; Table S4: Newcastle–Ottawa Scale (NOS) for cohort studies; Table S5: NOS-xs: adaptation of the NOS for cross-sectional studies. Figure S1: Leave-one-out sensitivity without Katz et al. (2005) [24] analysis for the meta-analysis of prognostic value of FMD to adverse events in HF patients with EF < 50%; Figure S2: Leave-one-out sensitivity without Meyer et al. (2005) [20] analysis for the meta-analysis of prognostic value of FMD to adverse events in HF patients with EF < 50%; Figure S3: Leave-one-out sensitivity without Paine et al. (2016) [4] analysis for the meta-analysis of prognostic value of FMD to adverse events in HF patients with EF < 50%; Figure S4: Leave-one-out sensitivity without Shechter et al. (2009) [25] analysis for the meta-analysis of prognostic value of FMD to adverse events in HF patients with EF < 50%; Figure S5: Leave-one-out sensitivity without Takishima et al. (2012) [10] analysis for the meta-analysis of prognostic value of FMD to adverse events in HF patients with EF < 50%; Figure S6: Leave-one-out sensitivity without Tarro Genta et al. (2013) [9] analysis for the meta-analysis of prognostic value of FMD to adverse events in HF patients with EF < 50%; Figure S7: Leave-one-out sensitivity analysis without Akiyama et al. (2012) [26] for the meta-analysis of prognostic value of RHI to adverse events in HF patients; Figure S8: Leave-one-out sensitivity analysis without Kim et al. (2023) [28] for the meta-analysis of prognostic value of RHI to adverse events in HF patients; Figure S9: Leave-one-out sensitivity analysis without Paine et al. (2016) [4] for the meta-analysis of prognostic value of RHI to adverse events in HF patients.

## Abbreviations

The following abbreviations are used in this manuscript:

6MWT	6 min walking test
ACE	Angiotensin-converting enzyme
BNP	B-type natriuretic peptide
CHF	Chronic heart failure
CI	Confidence interval
CPET	Cardiopulmonary exercise testing
CRT	Cardiac resynchronization therapy
EF	Ejection fraction
FMD	Flow-mediated dilation
HF	Heart failure
HFpEF	HF with preserved ejection fraction
HFrEF	HF with reduced ejection fraction
HR	Hazard ratio
LV	Left ventricle
LVAD	LV assist device
LVEF	LV ejection fraction
LVRR	LV reverse remodeling
MACE	Major adverse cardiac events
MPHR	Maximal predicted heart rate
N/A	Not available
NO	Nitric oxide
NOS	Newcastle-Ottawa Scale
NT-pro-BNP	N-terminal pro-B-type natriuretic peptide
NYHA	New York Heart Association
OMT	Optimal medical therapy
Peak VO2	Peak oxygen consumption
PRISMA	Preferred Reporting Items for Systematic reviews and Meta-Analyses
PSR	Peak shear rate
RHI	Reactive hyperemia index
RH-PAT	Reactive hyperemia–peripheral arterial tonometry
SGLT-2	Sodium-glucose transport 2
UNOS	United network for organ sharing
USA	United States of America

## Appendix A

**Table A1:** Hazard ratio calculation using formula from Hebert et al. (2022) [17].

No	Raw Data	Meyer et al. (2005) [20]	Shechter et al. (2009) [25]
4	R_r_ (Total number of patients: exposed group)	38	41
5	R_c_ (Total number of patients: control group)	37	41
7	O_r_ (#deaths reported: exposed group)	20	22
8	O_c_ (#deathsreported: control group)	7	8
9	Log-Rank *p*-value (Kmor or Cox PH)	0.0033	0.01
	Calculation		
11	Estimated death rate: exposed group	0.526316	0.536585
12	Estimated death rate: control group	0.189189	0.195122
13	Difference in est. death rate (r − c)	0.337127	0.341463
14	Direction of difference (enter: 1 if D13 is positive or −1 if D13 is negative	1	1
16	Equation (*V*) = Vr = (O_total_ × R_r_ × R_c_)/(R_r_ + R_c_)^2^	6.7488	7.5
17	Equation (*VI*) = Variance Ln(HR) = 1/V_r_	0.148174	0.133333
18	Equation (*VII*) = O_r_ − E_r_=(√(O_total_ × R_r_ × R_c_)/(R_r_ + R_c_)) × Φ^−1^ (1 − *p*-value/2) × (direction of difference)	7.633291	7.054199
19	Equation (*VIII*) = ln(HR) = (O_r_ − E_r_)/V_r_	1.131059	0.94056
21	HR = e^Ln(HR)^	3.098937	2.561415
22	95% CI LOWER = e^(Ln(HR)−1.96×√p(varianceLn(HR)))^	1.457303	1.252159
23	95% CI UPPER = e^(Ln(HR)+1.96×√p(varianceLn(HR)))^	6.589849	5.239629
