# Supplementary material for: Prognostic Value of Flow-Mediated Dilation and Reactive Hyperemia Index in Heart Failure: A Systematic Review and Meta-Analysis
Source: J Clin Med. 2025 Dec 24;15(1):149. doi: 10.3390/jcm15010149 (PMC12786865; doi:10.3390/jcm15010149)
Supplement: Supplementary file 1 [file jcm-15-00149-s001.zip › jcm-4024914-supplementary.pdf]

Supplementary Tables and Figures

Table S1. PRISMA Checklist (2020)

Section and Topic	Item #	Checklist item	Location where item is reported
**TITLE**			
Title	1	Identify the report as a systematic review.	1
**ABSTRACT**			
Abstract	2	See the PRISMA 2020 for Abstracts checklist.	1
**INTRODUCTION**			
Rationale	3	Describe the rationale for the review in the context of existing knowledge.	2
Objectives	4	Provide an explicit statement of the objective(s) or question(s) the review addresses.	2
**METHODS**			
Eligibility criteria	5	Specify the inclusion and exclusion criteria for the review and how studies were grouped for the syntheses.	3
Information sources	6	Specify all databases, registers, websites, organisations, reference lists and other sources searched or consulted to identify studies. Specify the date when each source was last searched or consulted.	3
Search strategy	7	Present the full search strategies for all databases, registers and websites, including any filters and limits used.	3, supplementary file
Selection process	8	Specify the methods used to decide whether a study met the inclusion criteria of the review, including how many reviewers screened each record and each report retrieved, whether they worked independently, and if applicable, details of automation tools used in the process.	3
Data collection process	9	Specify the methods used to collect data from reports, including how many reviewers collected data from each report, whether they worked independently, any processes for obtaining or confirming data from study investigators, and if applicable, details of automation tools used in the process.	3
Data items	10a	List and define all outcomes for which data were sought. Specify whether all results that were compatible with each outcome domain in each study were sought (e.g. for all measures, time points, analyses), and if not, the methods used to decide which results to collect.	3

	10b	List and define all other variables for which data were sought (e.g. participant and intervention characteristics, funding sources). Describe any assumptions made about any missing or unclear information.	3
Study risk of bias assessment	11	Specify the methods used to assess risk of bias in the included studies, including details of the tool(s) used, how many reviewers assessed each study and whether they worked independently, and if applicable, details of automation tools used in the process.	3
Effect measures	12	Specify for each outcome the effect measure(s) (e.g. risk ratio, mean difference) used in the synthesis or presentation of results.	4
Synthesis methods	13a	Describe the processes used to decide which studies were eligible for each synthesis (e.g. tabulating the study intervention characteristics and comparing against the planned groups for each synthesis (item #5)).	4
	13b	Describe any methods required to prepare the data for presentation or synthesis, such as handling of missing summary statistics, or data conversions.	4
	13c	Describe any methods used to tabulate or visually display results of individual studies and syntheses.	4
	13d	Describe any methods used to synthesize results and provide a rationale for the choice(s). If meta-analysis was performed, describe the model(s), method(s) to identify the presence and extent of statistical heterogeneity, and software package(s) used.	4
	13e	Describe any methods used to explore possible causes of heterogeneity among study results (e.g. subgroup analysis, meta-regression).	4
	13f	Describe any sensitivity analyses conducted to assess robustness of the synthesized results.	4
Reporting bias assessment	14	Describe any methods used to assess risk of bias due to missing results in a synthesis (arising from reporting biases).	3
Certainty assessment	15	Describe any methods used to assess certainty (or confidence) in the body of evidence for an outcome.	4
**RESULTS**			
Study selection	16a	Describe the results of the search and selection process, from the number of records identified in the search to the number of studies included in the review, ideally using a flow diagram.	4
	16b	Cite studies that might appear to meet the inclusion criteria, but which were excluded, and explain why they were excluded.	NA
Study characteristics	17	Cite each included study and present its characteristics.	6
Risk of bias in studies	18	Present assessments of risk of bias for each included study.	5, supplementary file

Results of individual studies	19	For all outcomes, present, for each study: (a) summary statistics for each group (where appropriate) and (b) an effect estimate and its precision (e.g. confidence/credible interval), ideally using structured tables or plots.	11
Results of syntheses	20a	For each synthesis, briefly summarise the characteristics and risk of bias among contributing studies.	11-12
	20b	Present results of all statistical syntheses conducted. If meta-analysis was done, present for each the summary estimate and its precision (e.g. confidence/credible interval) and measures of statistical heterogeneity. If comparing groups, describe the direction of the effect.	11-12
	20c	Present results of all investigations of possible causes of heterogeneity among study results.	11-12
	20d	Present results of all sensitivity analyses conducted to assess the robustness of the synthesized results.	11
Reporting biases	21	Present assessments of risk of bias due to missing results (arising from reporting biases) for each synthesis assessed.	5
Certainty of evidence	22	Present assessments of certainty (or confidence) in the body of evidence for each outcome assessed.	11
**DISCUSSION**			
Discussion	23a	Provide a general interpretation of the results in the context of other evidence.	12-14
	23b	Discuss any limitations of the evidence included in the review.	14
	23c	Discuss any limitations of the review processes used.	14
	23d	Discuss implications of the results for practice, policy, and future research.	14
**OTHER INFORMATION**			
Registration and protocol	24a	Provide registration information for the review, including register name and registration number, or state that the review was not registered.	3
	24b	Indicate where the review protocol can be accessed, or state that a protocol was not prepared.	3
	24c	Describe and explain any amendments to information provided at registration or in the protocol.	NA
Support	25	Describe sources of financial or non-financial support for the review, and the role of the funders or sponsors in the review.	15
Competing interests	26	Declare any competing interests of review authors.	15
Availability of data, code and other materials	27	Report which of the following are publicly available and where they can be found: template data collection forms; data extracted from included studies; data used for all analyses; analytic code; any other materials used in the review.	NA

Table S2. PRISMA Checklist for Abstract (2020)

Section and Topic	Item #	Checklist item	Reported (Yes/No)
**TITLE**			
Title	1	Identify the report as a systematic review.	Yes
**BACKGROUND**			
Objectives	2	Provide an explicit statement of the main objective(s) or question(s) the review addresses.	Yes
**METHODS**			
Eligibility criteria	3	Specify the inclusion and exclusion criteria for the review.	Yes
Information sources	4	Specify the information sources (e.g. databases, registers) used to identify studies and the date when each was last searched.	Yes
Risk of bias	5	Specify the methods used to assess risk of bias in the included studies.	No
Synthesis of results	6	Specify the methods used to present and synthesise results.	Yes
**RESULTS**			
Included studies	7	Give the total number of included studies and participants and summarise relevant characteristics of studies.	Yes
Synthesis of results	8	Present results for main outcomes, preferably indicating the number of included studies and participants for each. If meta-analysis was done, report the summary estimate and confidence/credible interval. If comparing groups, indicate the direction of the effect (i.e. which group is favoured).	Yes
**DISCUSSION**			
Limitations of evidence	9	Provide a brief summary of the limitations of the evidence included in the review (e.g. study risk of bias, inconsistency and imprecision).	No
Interpretation	10	Provide a general interpretation of the results and important implications.	Yes
**OTHER**			
Funding	11	Specify the primary source of funding for the review.	No
Registration	12	Provide the register name and registration number.	No

Table S3. Detailed search strategy for each database

Database	Keywords and boolean operator	Article(s) retrieved	Note
ScienceDirect	("flow-mediated dilation")AND("heart failure")AND("hospitalization" OR "mortality" OR "death" OR "quality of life" OR "symptoms" OR "outcomes")AND("patients")	982	Filter applied to restrict search on research articles only.
DOAJ	"flow-mediated dilation" OR "flow-mediated vasodilation" OR "endothelial function" AND "heart failure" OR "chronic heart failure" AND "hospitalization" OR "mortality" OR "quality of life" OR "outcomes"	51	
IEEE	("Document Title":flow-mediated dilation) OR ("Document Title":flow mediated vasodilation) OR ("Document Title":endothelial function) AND ("Document Title":heart failure) OR ("Document Title":chronic heart failure) AND ("Document Title":hospitalization) OR ("Document Title":mortality) OR ("Document Title":quality of life) OR ("Document Title":symptoms) OR ("Document Title":outcome)	593	Filter applied to restrict search on journal only.
PubMed	("flow-mediated dilation"[Title/Abstract] OR "flow mediated vasodilation"[Title/Abstract] OR "Endothelial function"[Title/Abstract]) AND ("heart failure"[MeSH Terms] OR "chronic heart failure"[Title/Abstract] OR "HFpEF"[Title/Abstract] OR "HFrEF"[Title/Abstract]) AND ("hospitalization"[Title/Abstract] OR "mortality"[Title/Abstract] OR "death"[Title/Abstract] OR "quality of life"[Title/Abstract] OR "symptoms"[Title/Abstract] OR "outcomes"[Title/Abstract]) AND ("patients"[Title/Abstract] OR "subjects"[Title/Abstract])	241	
EBSCO	(flow-mediated dilation or flow mediated vasodilation or vasodilation) AND (heart failure or chronic heart failure or HFpEF or HFrEF) AND (hospitalization or mortality or quality of life or outcomes) AND (subjects or patients)	1	

Web of Science	(((AB=("flow-mediated dilation" OR "flow-mediated vasodilation" OR "endothelial function")) AND AB=("heart failure" OR "chronic heart failure" OR "HFpEF" OR "HFrEF")) AND AB=("hospitalization" OR "mortality" OR "quality of life" OR "outcomes")) AND AB=("patients" OR "subjects")	273
Proquest	abstract("flow-mediated dilation" OR "flow-mediated vasodilation" OR "endothelial function") AND abstract("heart failure" OR "chronic heart failure" OR "HFpEF" OR "HFrEF") AND abstract("hospitalization" OR "mortality" OR "quality of life" OR "outcomes") AND abstract("patients" OR "subjects")	66
Scopus	TITLE-ABS-KEY ("flow-mediated dilation" OR "flow-mediated vasodilation" OR "endothelial function" AND "heart failure" OR "chronic heart failure" OR "HFpEF" OR "HFrEF" AND "hospitalization" OR "mortality" OR "quality of life" OR "outcomes" AND "patients" OR "subjects")	698
Cochrane	TitleAbstractKey "flow-mediated dilation" OR "flow-mediated vasodilation" OR "endothelial function" AND "heart failure" OR "chronic heart failure" OR "HFpEF" OR "HFrEF" AND "hospitalization" OR "mortality" OR "quality of life" OR "outcomes" AND "patients" OR "subjects"	311

Table S4. Newcastle-Ottawa Scale (NOS) for cohort studies

COHORT										
Journal	Selection				Comparability	Outcome			Total Score	RESULT
	Representativeness of the Exposed Cohort	Selection of the Non-Exposed Cohort	Ascertainment of Exposure	Demonstration That Outcome of Interest Was Not Present at Start of Study	Comparability of cohorts on the basis of the design or analysis	Assessment of outcome	Was follow-up long enough for outcomes to occur	Adequacy of Follow Up of Cohorts		
Akar et al. (2008)	*	*	*	*	*	*	*	*	**8**	Low risk of bias
Genta et al. (2013)	*	*	*	*	**	*	*	*	**9**	Low risk of bias
Katz et al. (2005)	*	*	*	*	*	*	*	*	**8**	Low risk of bias
Kihara et al. (2002)	*	*	*	*	-	*	*	*	**7**	Low risk of bias
Meyer et al. (2005)	*	*	*	*	*	*	*	*	**8**	Low risk of bias
Paine et al. (2016)	*	*	*	*	**	*	*	*	**9**	Low risk of bias
Poelzl et al. (2006)	*	*	*	*	**	*	*	*	**9**	Low risk of bias
Shechter et al. (2009)	*	*	*	*	**	*	*	*	**9**	Low risk of bias
Takishima et al. (2012)	*	*	*	*	**	*	*	*	**9**	Low risk of bias

Xie et al. (2016)	*	*	*	*	**	*	*	*	**9**	Low risk of bias
Fischer et al (2004)	*	*	*	*	*	*	*	*	**8**	Low risk of bias
Kim et al (2023)	*	*	*	*	**	*	*	*	**9**	Low risk of bias
Tsigkou et al. (2023)	*	*	*	*	**	*	*	*	**9**	Low risk of bias
Yufu et al (2015)	*	*	*	*	**	*	*	*	**9**	Low risk of bias
Akiyama et al (2012)	*	*	*	*	**	*	*	*	**9**	Low risk of bias

Table S5. NOS-xs: adaptation of the NOS for cross-sectional studies

Journal	Study Sample Selection		Assessment of exposure and outcome		Confounding factors		Total Score	RESULT
	Representativeness of the study size	Sample size	Assessment of the exposure(s)	Assessment of exposure and outcome(s)	Adjustment of confounder(s)	Assessment of confounder(s)		
Vittorio et al. (2010)	*	*	**	**	*	*	**8**	Low Risk of Bias
Haykowsky et al. (2012)	*	*	**	**	-	-	**6**	Moderate Risk of Bias

Study or Subgroup	log[Hazard Ratio]	SE	Weight	Hazard Ratio IV, Random, 95% CI
Katz 2005	0.1823	0.0779	0.0%	1.20 [1.03, 1.40]
Meyer 2005	1.1282	0.386	13.7%	3.09 [1.45, 6.58]
Paine 2016	0.0602	0.0343	31.3%	1.06 [0.99, 1.14]
Shechter 2009	0.94	0.3658	14.5%	2.56 [1.25, 5.24]
Takishima 2012	1.0986	0.4267	12.2%	3.00 [1.30, 6.92]
Tarro Genta 2013	0.3521	0.1146	28.3%	1.42 [1.14, 1.78]
**Total (95% CI)**			**100.0%**	**1.72 [1.19, 2.50]**

Heterogeneity: Tau² = 0.11; Chi² = 24.01, df = 4 (P < 0.0001); I² = 83%
Test for overall effect: Z = 2.86 (P = 0.004)

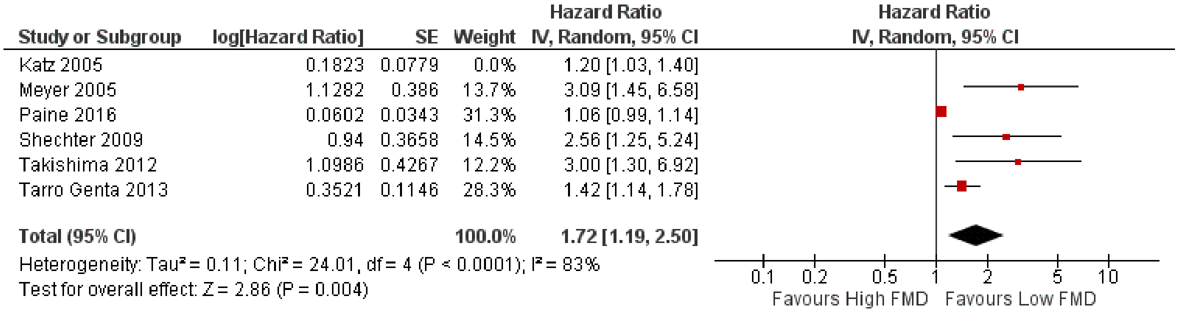

Figure S1. Leave-one-out sensitivity analysis without Katz et al. (2005) for the meta-analysis of prognostic value of FMD to adverse events in HF patients with EF <50%

Study or Subgroup	log[Hazard Ratio]	SE	Weight	Hazard Ratio IV, Random, 95% CI
Katz 2005	0.1823	0.0779	29.3%	1.20 [1.03, 1.40]
Meyer 2005	1.1282	0.386	0.0%	3.09 [1.45, 6.58]
Paine 2016	0.0602	0.0343	33.5%	1.06 [0.99, 1.14]
Shechter 2009	0.94	0.3658	6.9%	2.56 [1.25, 5.24]
Takishima 2012	1.0986	0.4267	5.4%	3.00 [1.30, 6.92]
Tarro Genta 2013	0.3521	0.1146	24.9%	1.42 [1.14, 1.78]
**Total (95% CI)**			**100.0%**	**1.33 [1.08, 1.64]**

Heterogeneity: Tau² = 0.03; Chi² = 17.95, df = 4 (P = 0.001); I² = 78%
Test for overall effect: Z = 2.65 (P = 0.008)

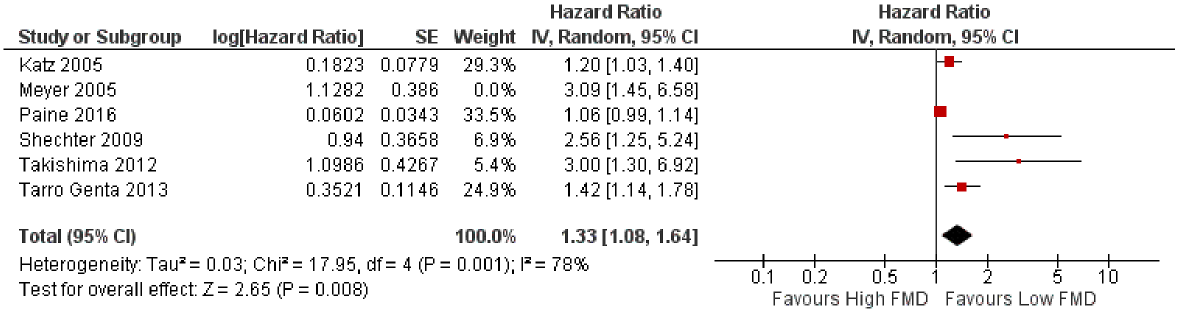

Figure S2. Leave-one-out sensitivity analysis without Meyer et al. (2005) for the meta-analysis of prognostic value of FMD to adverse events in HF patients with EF <50%

Study or Subgroup	log[Hazard Ratio]	SE	Weight	Hazard Ratio IV, Random, 95% CI
Katz 2005	0.1823	0.0779	33.8%	1.20 [1.03, 1.40]
Meyer 2005	1.1282	0.386	12.0%	3.09 [1.45, 6.58]
Paine 2016	0.0602	0.0343	0.0%	1.06 [0.99, 1.14]
Shechter 2009	0.94	0.3658	12.8%	2.56 [1.25, 5.24]
Takishima 2012	1.0986	0.4267	10.4%	3.00 [1.30, 6.92]
Tarro Genta 2013	0.3521	0.1146	31.0%	1.42 [1.14, 1.78]
**Total (95% CI)**			**100.0%**	**1.72 [1.25, 2.36]**

Heterogeneity: Tau² = 0.07; Chi² = 13.66, df = 4 (P = 0.008); I² = 71%
Test for overall effect: Z = 3.32 (P = 0.0009)

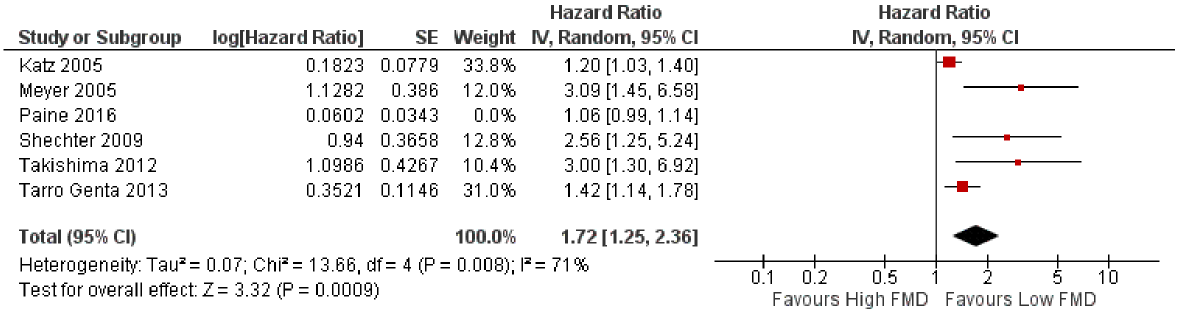

Figure S3. Leave-one-out sensitivity analysis without Paine et al. (2016) for the meta-analysis of prognostic value of FMD to adverse events in HF patients with EF <50%

Study or Subgroup	log[Hazard Ratio]	SE	Weight	Hazard Ratio IV, Random, 95% CI
Katz 2005	0.1823	0.0779	29.2%	1.20 [1.03, 1.40]
Meyer 2005	1.1282	0.386	6.9%	3.09 [1.45, 6.58]
Paine 2016	0.0602	0.0343	32.9%	1.06 [0.99, 1.14]
Shechter 2009	0.94	0.3658	0.0%	2.56 [1.25, 5.24]
Takishima 2012	1.0986	0.4267	5.8%	3.00 [1.30, 6.92]
Tarro Genta 2013	0.3521	0.1146	25.2%	1.42 [1.14, 1.78]
**Total (95% CI)**			**100.0%**	**1.35 [1.08, 1.69]**

Heterogeneity: Tau² = 0.04; Chi² = 19.76, df = 4 (P = 0.0006); I² = 80%
Test for overall effect: Z = 2.68 (P = 0.007)

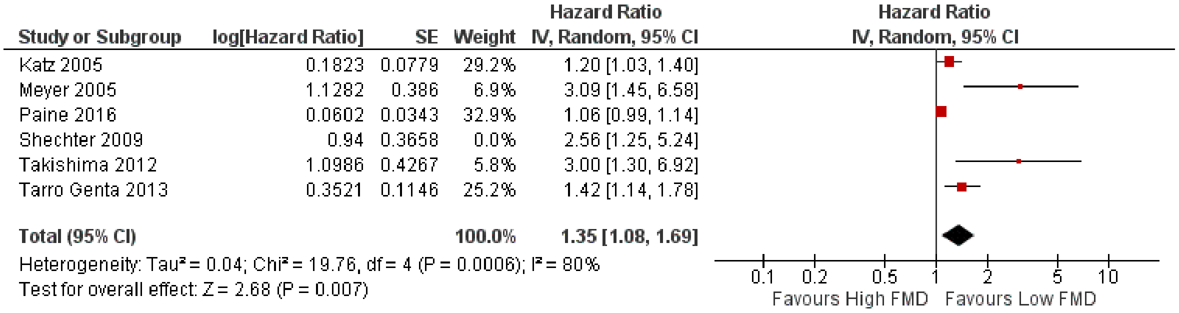

Figure S4. Leave-one-out sensitivity analysis without Shechter et al. (2009) for the meta-analysis of prognostic value of FMD to adverse events in HF patients with EF <50%

Study or Subgroup	log[Hazard Ratio]	SE	Weight	Hazard Ratio IV, Random, 95% CI
Katz 2005	0.1823	0.0779	28.8%	1.20 [1.03, 1.40]
Meyer 2005	1.1282	0.386	6.7%	3.09 [1.45, 6.58]
Paine 2016	0.0602	0.0343	32.5%	1.06 [0.99, 1.14]
Shechter 2009	0.94	0.3658	7.3%	2.56 [1.25, 5.24]
Takishima 2012	1.0986	0.4267	0.0%	3.00 [1.30, 6.92]
Tarro Genta 2013	0.3521	0.1146	24.7%	1.42 [1.14, 1.78]
**Total (95% CI)**			**100.0%**	**1.35 [1.09, 1.68]**

Heterogeneity: Tau² = 0.04; Chi² = 19.54, df = 4 (P = 0.0006); I² = 80%
Test for overall effect: Z = 2.72 (P = 0.007)

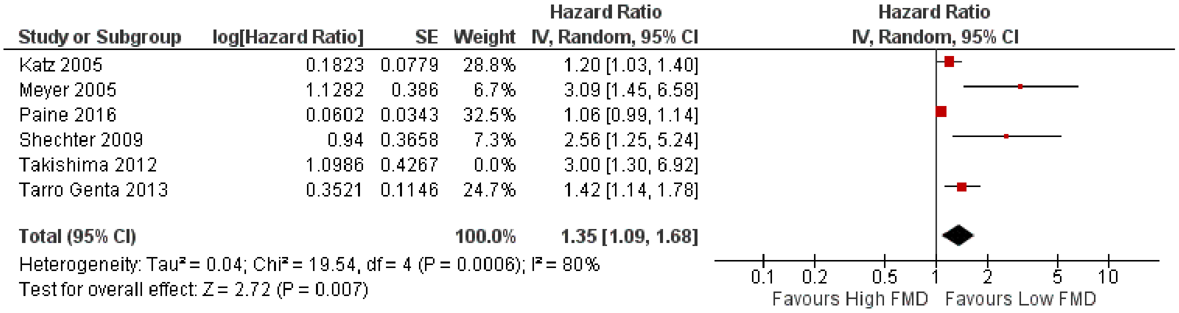

Figure S5. Leave-one-out sensitivity analysis without Takishima et al. (2012) for the meta-analysis of prognostic value of FMD to adverse events in HF patients with EF <50%

Study or Subgroup	log[Hazard Ratio]	SE	Weight	Hazard Ratio IV, Random, 95% CI
Katz 2005	0.1823	0.0779	33.9%	1.20 [1.03, 1.40]
Meyer 2005	1.1282	0.386	9.9%	3.09 [1.45, 6.58]
Paine 2016	0.0602	0.0343	37.0%	1.06 [0.99, 1.14]
Shechter 2009	0.94	0.3658	10.7%	2.56 [1.25, 5.24]
Takishima 2012	1.0986	0.4267	8.5%	3.00 [1.30, 6.92]
Tarro Genta 2013	0.3521	0.1146	0.0%	1.42 [1.14, 1.78]
**Total (95% CI)**			**100.0%**	**1.48 [1.12, 1.95]**

Heterogeneity: Tau² = 0.05; Chi² = 20.31, df = 4 (P = 0.0004); I² = 80%
Test for overall effect: Z = 2.76 (P = 0.006)

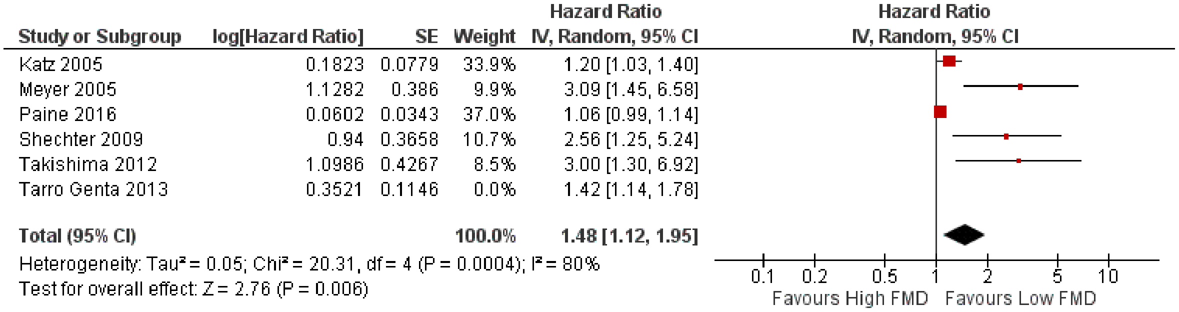

Figure S6. Leave-one-out sensitivity analysis without Tarro Genta et al. (2013) for the meta-analysis of prognostic value of FMD to adverse events in HF patients with EF <50%

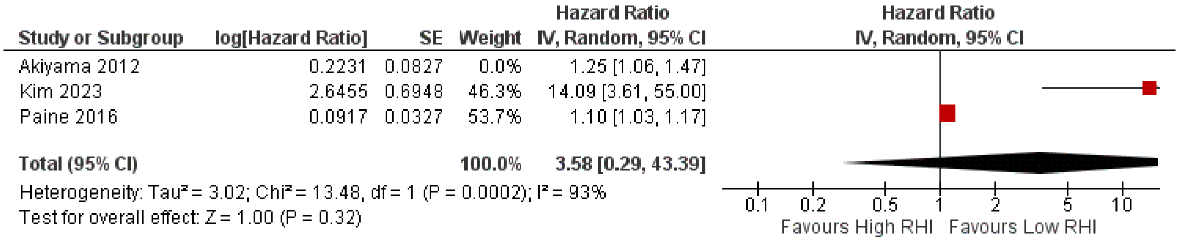

Figure S7. Leave-one-out sensitivity analysis without Akiyama et al. (2012) for the meta-analysis of prognostic value of RHI to adverse events in HF patients

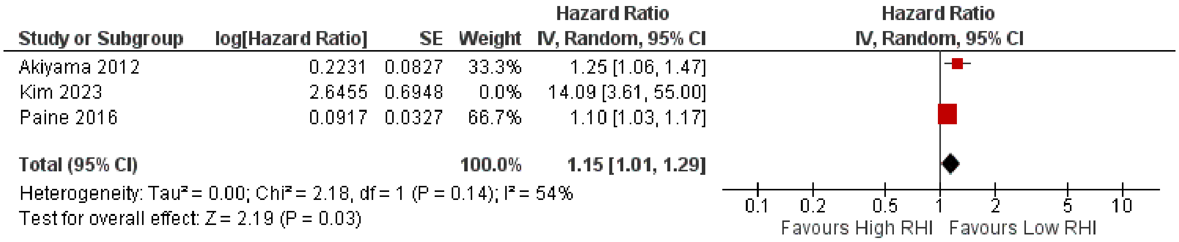

Figure S8. Leave-one-out sensitivity analysis without Kim et al. (2023) for the meta-analysis of prognostic value of RHI to adverse events in HF patients

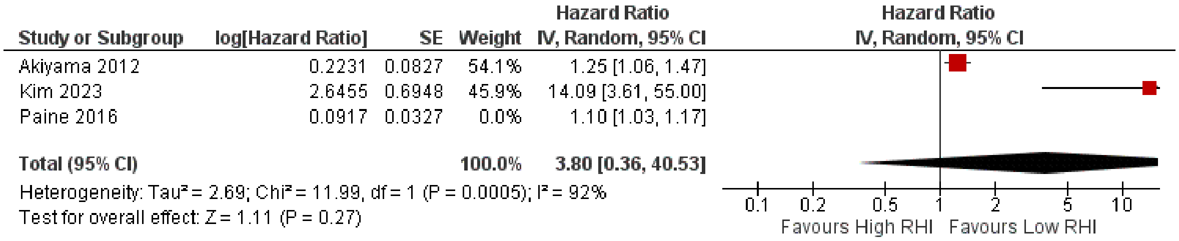

Figure S9. Leave-one-out sensitivity analysis without Paine et al. (2016) for the meta-analysis of prognostic value of RHI to adverse events in HF patients
